# Polarization-dependent optical responses in natural 2D layered mineral teallite

**DOI:** 10.1038/s41598-021-01511-z

**Published:** 2021-11-08

**Authors:** Ravi P. N. Tripathi, Xiaodong Yang, Jie Gao

**Affiliations:** 1 Department of Mechanical and Aerospace Engineering, Missouri University of Science and Technology, Rolla, MO 65409 USA; 2 Department of Mechanical Engineering, Stony Brook University, Stony Brook, NY 11794 USA

**Keywords:** Nonlinear optics, Two-dimensional materials

## Abstract

Multi-element layered materials enable the use of stoichiometric variation to engineer their optical responses at subwavelength scale. In this regard, naturally occurring van der Waals minerals allow us to harness a wide range of chemical compositions, crystal structures and lattice symmetries for layered materials under atomically thin limit. Recently, one type of naturally occurring sulfide mineral, ternary teallite has attained significant interest in the context of thermoelectric, optoelectronic, and photovoltaic applications, but understanding of light-matter interactions in such ternary teallite crystals is scarcely available. Herein, polarization-dependent linear and nonlinear optical responses in mechanically exfoliated teallite crystals are investigated including anisotropic Raman modes, wavelength-dependent linear dichroism, optical band gap evolution, and anisotropic third-harmonic generation (THG). Furthermore, the third-order nonlinear susceptibility of teallite crystal is estimated using the thickness-dependent THG emission process. We anticipate that our findings will open the avenue to a better understanding of the tailored light-matter interactions in complex multi-element layered materials and their implications in optical sensors, frequency modulators, integrated photonic circuits, and other nonlinear signal processing applications.

## Introduction

Nowadays, van der Waals (vdW) nanomaterials has been established as a promising platform to understand light-matter interactions at subwavelength scale and create building blocks for optoelectronic and nanophotonic applications, due to their strong quantum confinement effects, dangling-bond-free surface, interlayer coupling, and anisotropic physical responses^1–6^. Since the successful mechanical isolation of naturally occurring two-dimensional (2D) materials^7^, unprecedented efforts have been shown in thriving its prospect in the technological improvisation ranging from energy storage to optoelectronic applications^2, 5, 8, 9^. Noticeably, the initial phase of the explored 2D materials is almost confined to mono- and binary-element materials^10–14^, but with the rapid growth in material science and the demand to meet the high performance of future electronic and optoelectronic devices, extensive interests have been shown in exploring multi-element 2D materials^15, 16^. Owing to the diverse chemical compositions and crystal structures, complex multi-element layered materials facilitate an additional degree of freedom to tailor the physical, chemical, and optical responses^15–18^. Furthermore, in contrast to their mono- and binary-element counterparts, multi-element 2D materials have endorsed their prospect by demonstrating several fascinating phenomena such as linear dichroism transition^19, 20^, exceptional charge carrier mobility^21^, widely tunable band gap^22^, ultrafast lasing^23^, and infrared photodetection^24^. Despite this progress so far, rational and controllable chemical synthesis of multi-element layered materials remains a challenge.

As an alternate, naturally occurring layered minerals pave an interesting way to prepare multi-element ultrathin flakes via mechanical exfoliation^25, 26^. In recent years, variety of natural vdW minerals such as teallite^27^, franckeite^28, 29^ and getchellite^30^ have been explored. Among these, as one type of naturally occurring vdW sulfosalt mineral, teallite has recently attained significant interest for advanced optoelectronic applications^27, 31, 32^. Teallite is a lead–tin sulfosalt mineral with the ideal chemical formula of PbSnS_2_, which was first described in 1904 from Bolivia and named in honor of the geologist Jethro Justinian Harris Teall^33^. Teallite occurs as thin micaceous plates showing silvery-gray, lead-gray to iron-gray color and black streak. It has a Mohs scale hardness of 1.5 to 2 and the crystals are flexible and malleable. Ternary teallite is *p*-type semiconductor consists of Pb or Sn atoms bonded to S atoms and shaped as puckered bilayer structures similar to black phosphorus^32^. Also, owing to high light-absorption efficiency, teallite has been recognized as a potential candidate for solar cell applications^34^. Furthermore, the exfoliated teallite nanosheets are also explored for energy related applications such as thermoelectrics^35^ and supercapacitor^36^. However, there is no comprehensive study available on fundamental understanding of linear and nonlinear optical responses of teallite crystal yet. Moreover, these insights regarding the crystal structures and other microscopic processes are desirable for performance efficiency enhancement and engineering the application-based optical and optoelectronic responses for the improvisation of nanoscale devices.

Motivated with this, herein we explore the polarization-dependent anisotropic linear and nonlinear optical responses of mechanically exfoliated ternary teallite thin crystals. Thin teallite flakes are prepared via the mechanical exfoliation from natural bulk teallite mineral. The prepared teallite flakes are characterized using optical microscope, atomic force microscope (AFM), high-resolution transmission electron microscopy (HRTEM) and energy dispersive X-ray spectroscopy (EDXS) techniques to determine the crystal’s information, including flake shape, flake size, layer thickness, surface smoothness, atomic arrangement and chemical composition. Further, the flakes are probed spectroscopically to determine the crystalline axis and optical axis with polarization-resolved Raman spectroscopy and optical absorption spectroscopy. The optical absorption spectra are further analyzed to understand the angle-resolved evolution of optical band gap in teallite semiconductor. In last, anisotropic THG response of teallite crystal with respect to the incident linear polarization of pump beam is investigated and we estimate the third-order nonlinear susceptibility from the thickness-dependent THG emission process. The experimental results are corroborated by the theoretical models. Our results will lead to new insights in better understanding of light-matter interactions in complex multi-element vdW layered materials and advancing many photonics applications such as frequency modulators, integrated photonic circuits, and nonlinear optical signal processing.

## Results

### Sample preparation and characterization of teallite crystals

**Figure 1:**
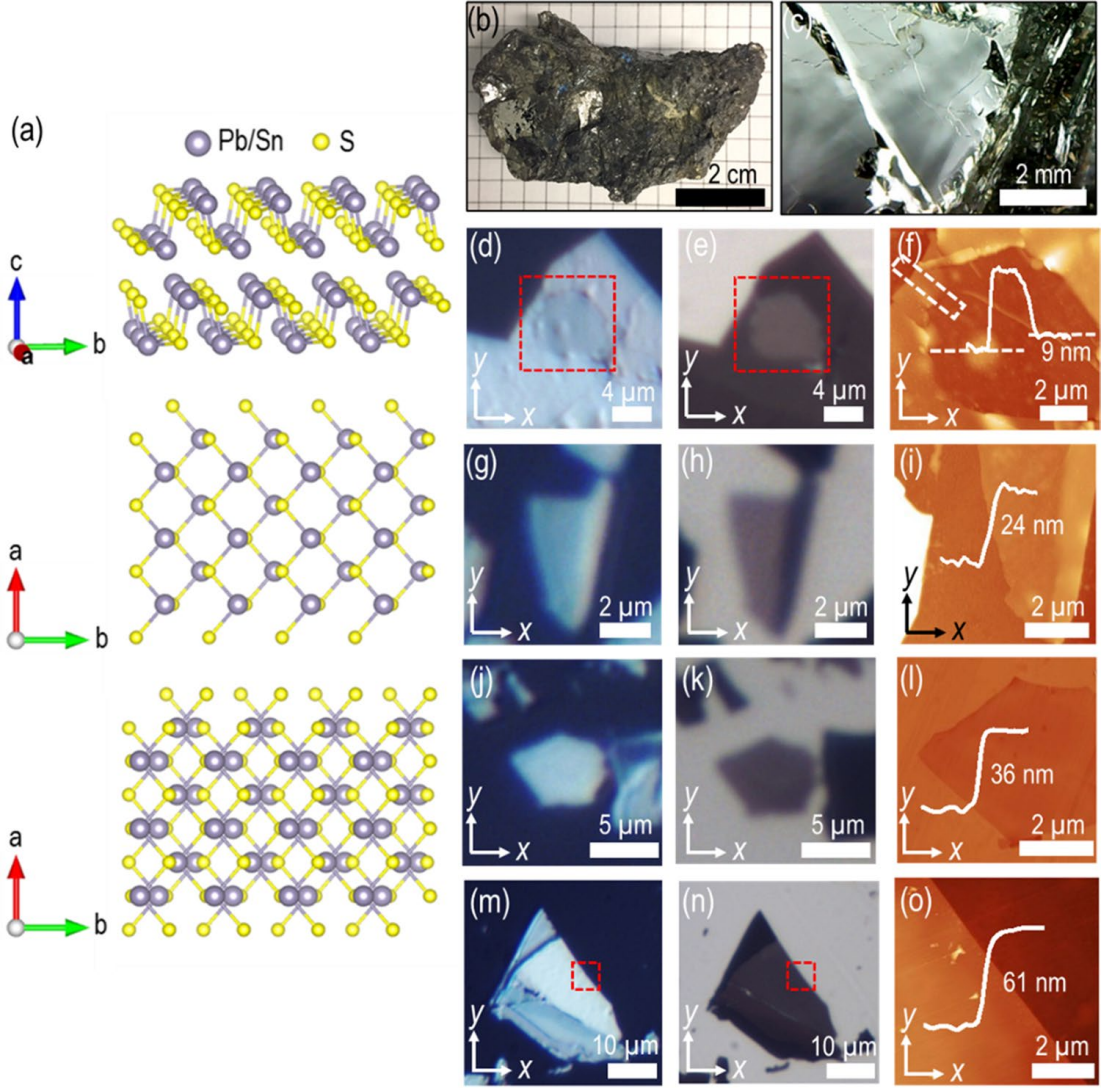
(**a**) Schematic representation of teallite crystal structure with the side view of puckered bilayer, the top view of single layer, and the top view of bilayer. (**b**) Image of bulk natural teallite mineral. (**c**) The magnified view of a small portion from the bulk crystal. (**d**,**g**,**j**,**m**) Optical microscope reflection images and (**e**,**h**,**k**,**n**) transmission images of mechanically exfoliated teallite flakes of 9, 24, 36 and 61 nm transferred on glass substrates. (**f**,**i**,**l**,**o**) The corresponding AFM images of these flakes or sections of flakes as shown with red square boxes in (**d**,**e**,**m**,**n**). The AFM line profiles signify the thickness and surface smoothness of the scanned flakes.

Figure 1a shows the simplified view of the teallite crystal structure. As presented in Fig. 1a, teallite forms a puckered bilayer structure similar to that of black phosphorus and herzenbergite SnS with S atoms bonded to Pb or Sn atoms, where the second layer is rotated relative to the first layer by 180° according to the AB stacking order in the unit cell bilayer. It belongs to the orthorhombic crystal system with space group *Pnma* (No. 62) with the unit cell dimensions of *a* = 4.09 Å, *b* = 4.26 Å, *c* = 11.41 Å and *α* = *β* = *γ* = 90°. The *a* and *b* axes correspond to the zigzag and armchair directions, with the *c*-axis perpendicular to the *a*-*b* plane and $\alpha$, $\beta$ and $\gamma$ are the angles between *a-*, *b-* and *c*-axis. Figure 1b shows a picture of a teallite mineral rock, where silvery-gray tightly packed micaceous foliated plates of teallite crystals are presented. The magnified view of a section is shown in Fig. 1c. We prepare the teallite thin flakes of various thicknesses on glass substrate via the mechanical exfoliation of the bulk natural teallite mineral (Monserrat, Oruro, Bolivia) using Nitto tape (SPV 224) and Scotch tape. Figure 1d,e,g,h,j,k,m,n show the optical microscope reflection and transmission images of several prepared teallite flakes with the thicknesses of 9, 24, 36 and 61 nm, respectively. Further, these teallite flakes are scanned using AFM to estimate the thickness and surface roughness. The captured AFM images are shown in Fig. 1f (9 nm), 1i (24 nm), 1l (36 nm), and 1o (61 nm), respectively.

**Figure 2:**
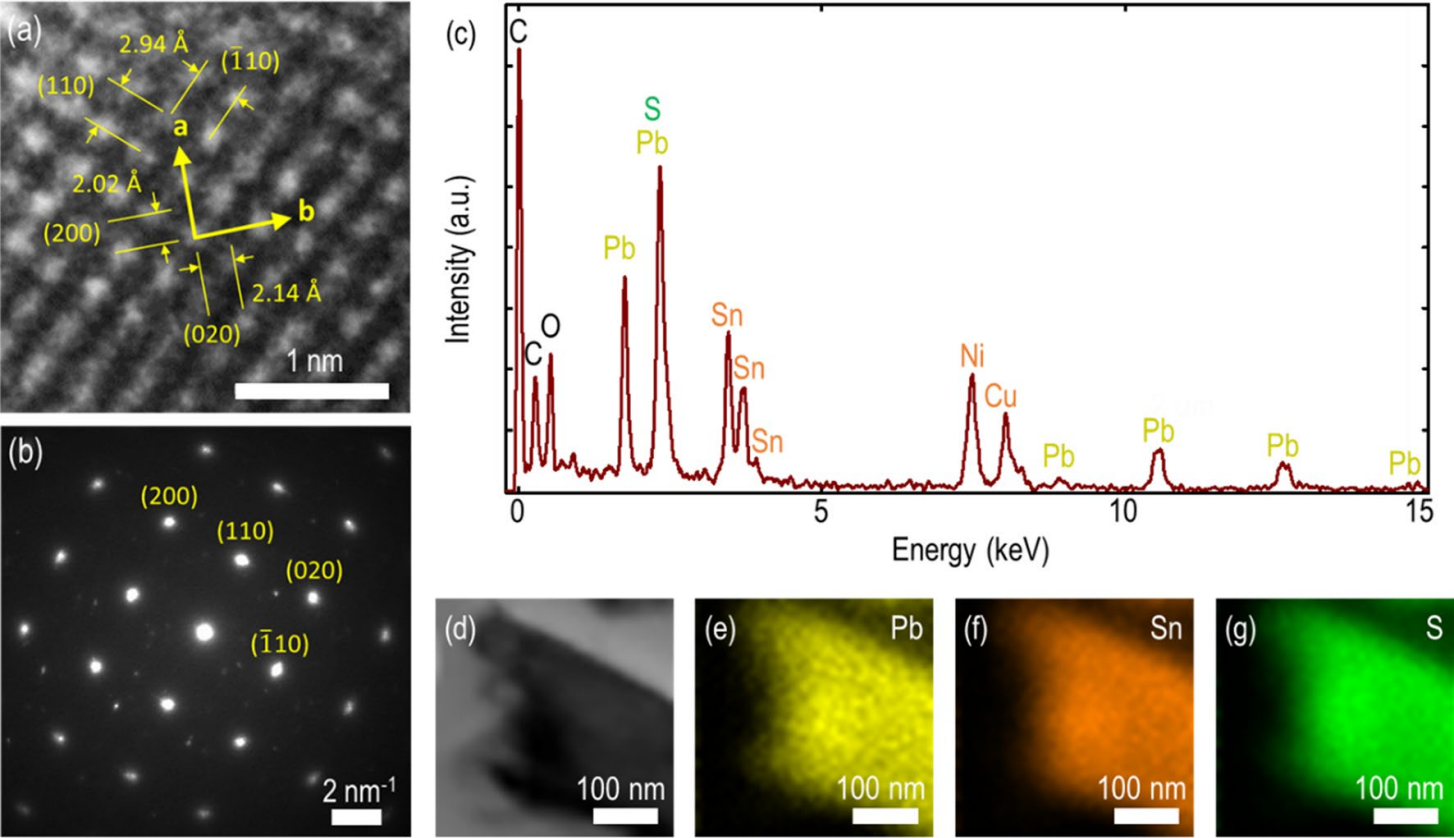
(**a**) Representative atomic HRTEM image of a thin teallite crystal. (**b**) The corresponding SAED pattern. (**c**) Recorded average EDXS spectrum. Three prime elements (Pb, Sn and S) as per their atomic weight percentage are considered for determining the chemical composition of teallite crystal. (**d**) Bright-field TEM image of the scanned region. (**e**–**g**) TEM-EDXS mapping of individual elements, emphasizing the consistency and presence of lead, tin, and sulfur in the probed teallite crystal.

Next, the structural information and chemical composition of teallite crystal are determined using HRTEM and EDXS characterization techniques. Figure 2a shows a HRTEM image of teallite crystal with the determined lattice spacing of 2.02 Å and 2.14 Å along the zigzag direction (*a*-axis) and the armchair direction (*b*-axis), with the intersection angle of around 90°. These spacing are found consistent with the (200) and (020) sets of planes for the orthorhombic teallite crystal. Both (110) and ($\overline{1 }$10) sets of planes have the lattice spacings of 2.94 Å with the intersection angle of around 86°, and both sets of planes have the angle of around 43° with respect to the (020) planes. Further, the recorded selected area electron diffraction (SAED) pattern is displayed in Fig. 2b, where the spot patterns are captured from the surface normal to the [001] crystal zone axis. The observed main reflection pattern is consistent with the previously reported pure teallite crystal structure^32^. This consistency underlines that the investigated crystal is of high quality and the crystalline nature or purity of the investigated crystal is not affected. Besides, we also observe few weak-intensity reflections in addition to the main reflections. We ascribe that these weak-intensity reflections are due to the slight distortion and modulation in actual teallite structure. Such minor distortion and modulation of the lattice parameters, unit cell dimensions and lattice symmetries in the resultant teallite crystal can be expected due to the geological origin of the naturally grown teallite mineral and the dual phase coexistence of herzenbergite-teallite. Subsequently, the chemical composition of ternary teallite crystal is quantified. Figure 2c shows the collected average EDXS spectrum, while the bright-field TEM image of the scanned region and the corresponding individual elemental mappings are shown in Fig. 2d–g. These recorded mappings emphasize the homogenous distribution of prime constituent elements of lead (Pb), tin (Sn) and sulfur (S). The obtained average elemental composition (as per atomic weight %) is summarized in Table 1, which is used to perform the compositional stoichiometry analysis. The resultant empirical formula is found to be Pb_0.58_Sn_1.58_S_2.00_ with the Pb:Sn ratio of 0.367. The obtained formula is not the excellent match with the generic teallite chemical formula PbSnS_2_ and the range of the chemical composition of constituent elements are significantly broad. These two factors emphasize the natural occurrence of herzenbergite-teallite series in the probed specimen^37^. It is noteworthy that the obtained formula lies within the variation range in chemical composition between Pb_0.428_Sn_1.572_S_2_ and Pb_0.784_Sn_1.216_S_2_ according to the chemical analysis of a series of herzenbergite-teallite minerals from Bolivia^38^, where the Pb:Sn ratio is from 0.272 to 0.645. The appearance of nickel (Ni) peak in the EDXS spectrum is ascribed to the TEM grid. Besides, the presence of small amounts of additional elements such as copper (Cu), carbon (C) and oxygen (O) in the recorded EDXS spectrum can be attributed to the mineral geological origins^25, 27, 37^. In addition, the high contribution of carbon (C) and oxygen (O) can be ascribed to the sample processing for TEM analysis and the underlying carbon support film of TEM grid. We used polymethyl methacrylate (PMMA)-assisted wet transfer method for transferring the mechanically exfoliated teallite flakes from glass substrate to TEM grid, which further leads to the accumulation of carbon-based adsorbates during the flake transfer^39^. Additionally, the recorded Raman modes of the teallite mineral sample are also compared with the Raman modes of the previously reported chemically synthesized pure teallite crystal to further elucidate any significant influence of these doped elements on the intrinsic and optical properties of teallite mineral. We found a good agreement between the observed Raman modes of the teallite mineral and those of the chemically synthesized highly pure teallite crystal. Thus, we conclude that the probed teallite mineral crystal is substantially pure, and the influence of the geological origin induced impurities on the optical responses would be minimal.

**Table 1:** EDXS quantification of bulk teallite crystal.

Elements	Concentration (atomic weight %)
Pb	14.00 ± 1.68
Sn	37.90 ± 1.65
S	48.10 ± 2.57

### Characterization of anisotropic Raman modes and determination of crystal axis in teallite crystals

**Figure 3:**
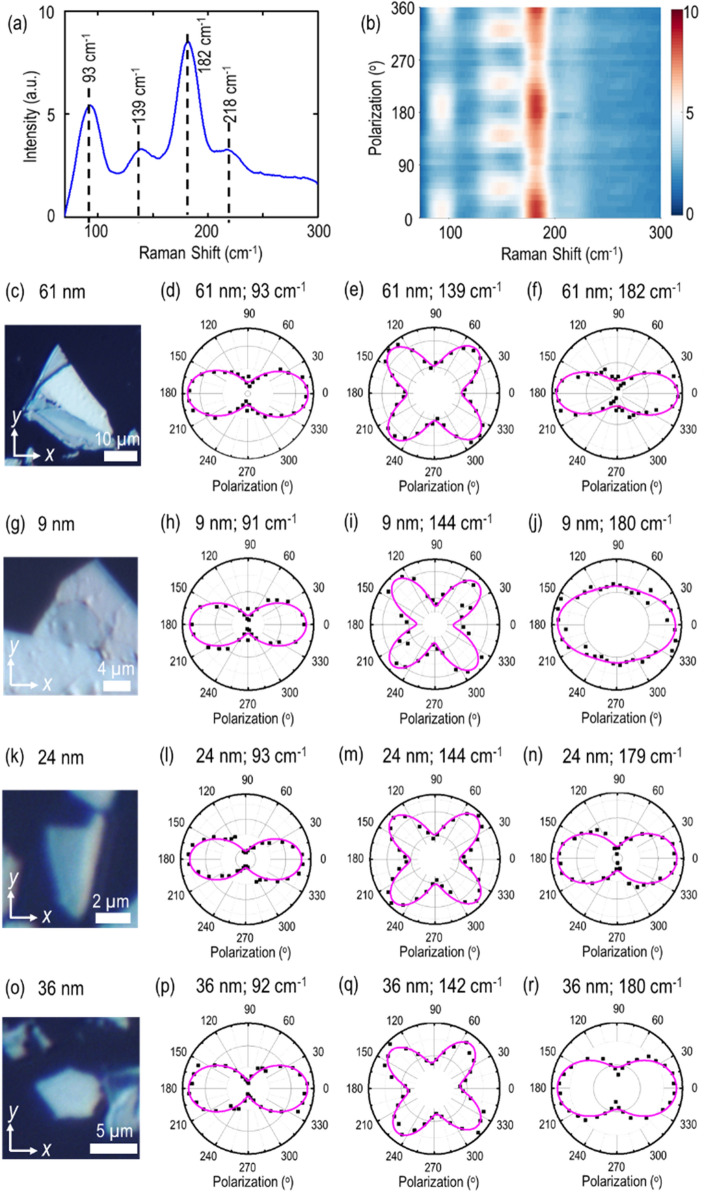
(**a**) Collected Raman spectrum for the 61 nm-thick teallite flake. The observed Raman modes are indicated with vertical black dashed lines. (**b**) Color map of angle-resolved Raman spectra in parallel polarization configuration for the 61 nm flake. (**c**,**g**,**k**,**o**) Captured optical microscope reflection images of the investigated 61, 9, 24 and 36 nm thick teallite crystals. (**d**–**f**) Polar plots of Raman intensities for different Raman modes at 93, 139 and 182 cm^−1^ in parallel polarization configuration for the 61 nm flake. (**h**–**j**,**l**–**n**,**p**–**r**) Polar plots of Raman modes for other teallite flakes of 9, 24 and 36 nm. Black squares are the experimental data, whereas magenta solid lines indicate the theoretical fits.

Next, the teallite flakes are characterized with polarization-resolved Raman spectroscopy to identify the crystal’ axes. The flake is illuminated with a 632.8 nm He–Ne laser. The desired input linear polarization is obtained by using a combination of a linear polarizer and a half-wave plate in the excitation path, while an analyzer is engaged in the collection path set to be in the parallel direction with respect to the input beam polarization. Figure 3a shows the recorded Raman spectrum from the 61 nm-thick teallite crystal in parallel polarization configuration, showing a series of intense Raman vibrational modes at 93, 139, 182 and 218 cm^−1^. Importantly, there are some similarities between the Raman spectra of teallite and herzenbergite SnS^40, 41^. The Raman peaks of 93, 182 and 218 cm^−1^ are assigned to the A_g_ modes, which are originated from the waving mode, the breathing mode, and the NaCl-type vibrational mode, respectively. While the peak of 139 cm^−1^ corresponds to the B_3g_ mode, which is attributed to the NaCl-type vibration along the zigzag direction. To gain further insight of the angular periodicity of these Raman modes, polarization-dependent Raman spectra are studied. Figure 3b shows the color map of the Raman intensity spectrum as per the input linear polarization angle in parallel polarization configuration, where the anisotropic nature of these Raman vibrational modes can be clearly observed. Figure 3c shows the optical microscope reflection image of the 61 nm-thick flake, and the corresponding polar plots of the Raman intensities of two A_g_ modes at 93 and 182 cm^−1^, one B_3g_ mode at 139 cm^−1^ are displayed in Fig. 3d–f, which are theoretically fitted using the following equations^32, 42^,

1 $${I}_{{A}_{g}}^{//}\propto {\left(\left|a\right|{\sin}^{2}\theta +\left|b\right|\cos{\varnothing }_{ba}{\cos}^{2}\theta \right)}^{2}+{\left(\left|b\right|\sin{\varnothing }_{ba}{\cos}^{2}\theta \right)}^{2}$$

2 $${I}_{{B}_{g}}^{//} \propto {\left|e\right|}^{2} {\sin}^{2}2\theta$$

where // denotes the parallel polarization configuration, *a*, *b* and *e* are the amplitudes of Raman tensor elements, and ${\varnothing }_{ba}= {\varnothing }_{b}- {\varnothing }_{a}$ denotes the associated phase difference between *a* and *b* elements. Further, we ensure the consistency of the observed angle-resolved Raman modes for teallite crystals with different thicknesses. Figure 3g,k,o show the optical reflection images of the investigated 9, 24 and 36 nm-thick flakes, and the corresponding A_g_ and B_3g_ modes are also plotted in Fig. 3h–j,l–n,p–r, respectively. The experimental data are denoted as black squares, whereas the theoretical fitting curves are shown with magenta solid lines, indicating a good agreement with each other. It is found that all the A_g_ modes show anisotropic two-lobe patterns with the maxima at 0° and 180°, whereas the B_3g_ mode shows anisotropic four-lobe pattern with the maxima at 45°, 135°, 225° and 315°. Furthermore, the orientation of A_g_ mode pattern indicates the crystal axis of the probed teallite flake, where *θ* = 0° direction (along *x*-axis) and *θ* = 90° direction (along *y*-axis) are accredited as the *b*-axis (armchair direction) and *a*-axis (zigzag direction) of teallite crystal, respectively.

### Polarization-resolved optical absorption and band gap evolution in teallite crystals

**Figure 4:**
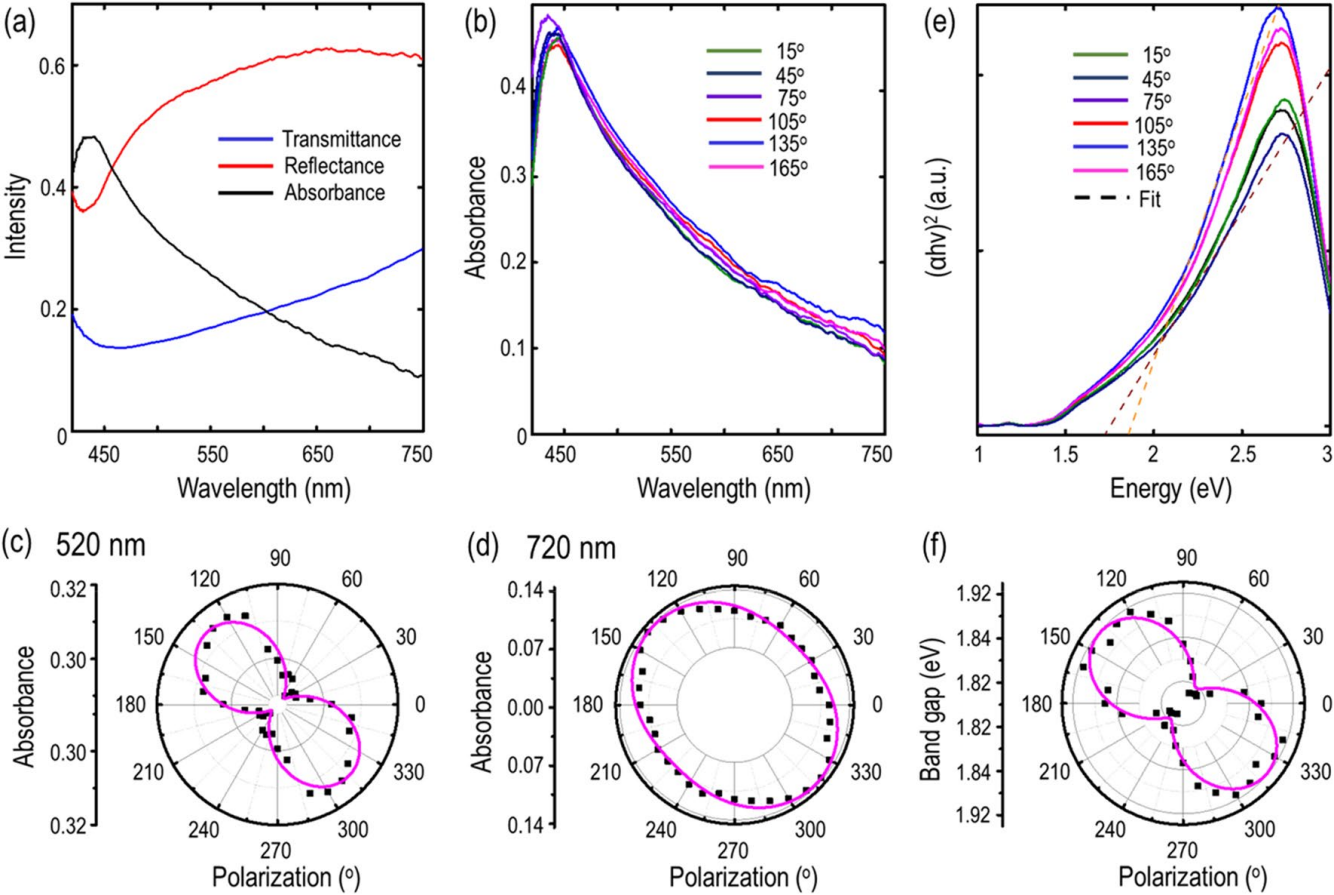
(**a**) Recorded reflectance (*R*), transmittance (*T*) and absorbance (*A*) spectra for the 61 nm- thick teallite flake. (**b**) Evolution of angle-resolved optical absorbance spectra. (**c**,**d**) Polar plots of absorbance at two different wavelengths of 520 nm and 720 nm. (**e**) Evolution of angle-resolved Tauc plots and extraction of the direct optical band gap in teallite crystal. (**f**) Polar plot of polarization-dependent optical band gap. In all the polar plots, the measured values are shown with black squares, whereas solid magenta lines signify the theoretical fits.

**Figure 5:**
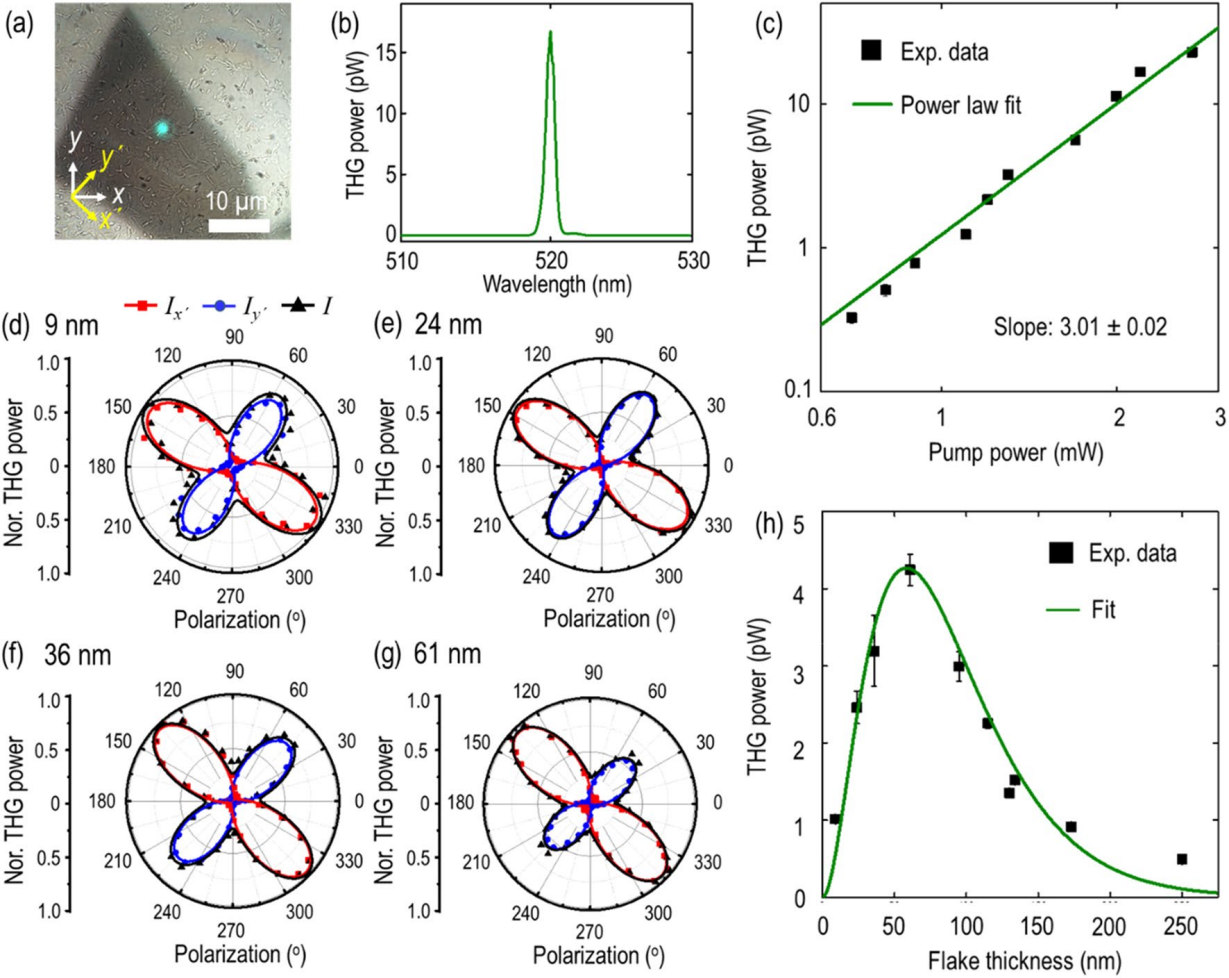
(**a**) Optical transmission microscope image of the 61 nm-thick teallite crystal with green THG emission. *x*- and *y*-axes indicate the crystalline *b*- and *a*-axes, whereas *x*′- and *y*′- axes denote the optical axis of the probed crystal and its perpendicular direction. (**b**) Recorded THG emission spectrum. (**c**) Log-scale plot between THG emission power and pump power. (**d**–**g**) Polarization-resolved THG emission power for four different teallite flakes with thicknesses of 9, 24, 36, and 61 nm. Red squares, blue dots and black triangles denote the measured data points along *x*′-component (${I}_{x^{\prime}}$), *y*′-component (${I}_{y^{\prime}}$), and total ($I$) THG emission power, respectively, while the theoretical fits are specified in the respective colors. (**h**) Thickness-dependent THG emission power. The measured THG emission power in (**c**) and (**h**) are shown with black squares, whereas the theoretical fits are indicated with solid green curves.

The reduced in-plane lattice symmetry and the prevalent anisotropic Raman vibrational modes of teallite crystal also endorse the presence of linear dichroism. To get further insight, we probe the optical absorption characteristics of the 61 nm flake within the visible frequency regime from 420 to 750 nm using polarization-resolved optical absorption spectroscopy. Figure 4a shows the measured reflectance (*R*), transmittance (*T*) and absorbance (*A* = 1 – *R* – *T*) spectra, keeping the input linear polarization fixed along the optical axis of the crystal in the [$\overline{1 }$10] lattice direction. Both the reflectance and transmittance spectra show an initial resonance dip around the wavelength of 450 nm and then gradually increase within the range of 450 nm to 750 nm. Also, the reflectance is higher as compared to the transmittance all over the visible frequency range. Accordingly, the absorbance spectrum shows a peak around 450 nm and then decreases up to the wavelength of 750 nm. To further examine the anisotropic features of optical absorption and linear dichroism, angle-resolved absorbance spectra are systematically measured for the 61 nm-thick teallite crystal, as shown in Fig. 4b. The input linear polarization angle is set relative to the *b*-axis of the crystal. Figure 4c,d show the polar plots of absorbance as per incident linear polarization angle at two different wavelengths of 520 nm and 720 nm, exhibiting anisotropic two-lobe patterns with the maxima at around − 43° and 137°. It is inferred that the teallite crystal anisotropically absorbs photons with the preference along the optical axis in the [$\overline{1 }$10] lattice direction, which is around 43° from the armchair direction along the *b*-axis. The optical axis of teallite crystal is indicated as the *x*′-axis in Fig. 5a. The experimentally measured data points are theoretically fitted using the equation $A \left(\theta \right)= {A}_{x^{\prime}} {\cos}^{2}\left(\theta \right)+ {A}_{y^{\prime}} {\sin}^{2}\left(\theta \right)$, where ${A}_{x^{\prime}}$ and ${A}_{y^{\prime}}$ are the absorbance magnitudes along the optical axis and its perpendicular direction. The absorption anisotropic ratio ${A}_{x^{\prime}}/{A}_{y^{\prime}}$ is obtained as 1.08 and 1.29 at 520 nm and 720 nm, respectively, showing that the teallite crystal exhibits strong wavelength-dependent linear dichroism effect.

We understand such lattice direction preferentiality for optical absorption in the context of the distorted lattice symmetry due to the structural complexity of teallite crystal compared to the standard herzenbergite SnS crystal. It is still unknown about how the Pb and Sn elements are exactly distributed in ideal PbSnS_2_ crystal yet. It has been studied that the Pb element alloying into SnS lattice can induce the sizable structural deformation of the in-plane lattice for PbSnS_2_ crystal, where the inclusion of 20% Pb into SnS leads to the lattice parameter *a* along the zigzag direction increases by 2% and the lattice parameter *b* along the armchair direction decreases by 1%^31^. Furthermore, there are four distinct structural phases commonly occurring in PbSnS_2_ crystal with different layer stacking sequences along the *c*-axis and different distances between the successive layers. All these structural phases for describing the PbSnS_2_ crystal are orthorhombic with the same unit cell dimensions, but exhibit differing lattice symmetry due to different Pb and Sn atom arrangement and ordering^43^. Therefore, the sizable structural deformation of the in-plane lattice and the lattice symmetry distortion of teallite crystal induced by the Pb alloying into SnS will affect the optical responses. In addition, the dual phase coexistence of herzenbergite-teallite in the natural mineral will further play a crucial role in modulating the lattice parameters, unit cell dimensions and the associated lattice symmetry in the resultant teallite crystal^37^. As shown in the EDXS analysis, the Pb:Sn ratio in our sample varies from 0.321 to 0.421. Such broad range variations in Pb:Sn ratio validate the coexistence of the herzenbergite-teallite phase and the imperfect miscibility in the natural mineral^37^. As a result, the investigated natural teallite crystals have more complicated distorted unit cell dimensions and lattice symmetries in contrast to the ideal PbSnS_2_ crystal structure, which will further affect the optical responses and may lead to the maximum optical absorption along the optical axis in the [$\overline{1 }$10] lattice direction, rather than the armchair direction along the *b*-axis. Nevertheless, further insight of these optical processes in stoichiometric controlled synthesis of complex 2D materials are inevitable to comprehensively explore such intriguing phenomena.

Moreover, such stoichiometric variation in these multi-element 2D materials could also strongly modulate the electronic band structure and optical band gap of the material. Hence, as a ternary-element semiconductor, teallite is an interesting specimen to explore the polarization-dependent evolution of optical band gap. The optical band gap of teallite crystal is estimated by the Tauc plot using the relation ${\left(\alpha h\nu \right)}^{n}=C(h\nu -{E}_{g})$^34^, where ${E}_{g}$ is optical band gap energy, $\alpha$ is the absorption coefficient corresponding to the photon energy $h\nu$ ($h$ is the Plank’s constant and $\nu$ is the frequency of the incident photon), $C$ is a constant and $n=2$, $1/2$, $2/3$ indicate an allowed direct transition, an allowed indirect transition, and a forbidden transition, respectively. The value of $\alpha$ can be extracted from the measured reflectance and transmittance spectra shown in Fig. 4a with the flake thickness $d$ using the following equation^44^

3 $$\alpha =\frac{1}{d} \ln\left\{\frac{2T}{{\left[{T}^{2}-{\left(1-R\right)}^{2}\right]+\left\{{\left[{T}^{2}-{\left(1-R\right)}^{2}\right]}^{2}+4{T}^{2}\right\}}^{1/2}}\right\}$$

Figure 4e shows the angle-resolved plot of ${\left(\alpha h\nu \right)}^{2}$ as a function of the photon energy $h\nu$ for the 61 nm-thick teallite crystal. The analysis of the Tauc plots in between 2.201 eV and 2.594 eV energy region gives a good linear fit for $n=2$, which underlines the allowed direct energy transition and the presence of direct optical band gap close to 1.87 eV. This value shows a decent match with the previously reported band gap for PbSnS_2_ thin films^32,34^. Interestingly, these extracted values of direct band gap from the Tauc plots show a systematic variation with the incident linear polarization angle. Figure 4f show the polar plot of optical band gap evolution with respect to the incident linear polarization angle. It is observed that the optical band gap of teallite crystal exhibits an anisotropic two-lobe pattern with the maximum value of 1.92 eV along the optical axis in the [$\overline{1 }$10] lattice direction and the minimum value of 1.83 eV along its perpendicular direction, which is strongly influenced with the structural symmetry in such complex 2D materials.

### Anisotropic THG response and third-order nonlinear susceptibility in teallite crystals

In last, we explore the effect of the low in-plane crystal symmetry of teallite crystal on the nonlinear optical response with polarization-resolved THG emission. The anisotropic THG emission is investigated with a 1560 nm pulse laser source with the spot size of 1.5 µm. Figure 5a shows the transmission microscope image with the green THG emission from the 61 nm-thick teallite flake. The corresponding THG emission spectrum is shown in Fig. 5b, showing a peak at 520 nm which is exactly one third of the pump wavelength. To further reaffirm the THG process, the log-scale plot of THG emission power as a function of the incident pump power is shown in Fig. 5c, in which the cubic power law fit endorses the THG process. Next, the in-plane anisotropic THG emission as a function of the incident pump beam polarization in different teallite flakes are measured. The incident pump polarization is controlled by engaging the combination of a linear polarizer and a half-wave plate in the excitation path. It is worth noting that the anisotropic total THG emission pattern is symmetrical with respect to the optical axis along the *x*′-axis and its perpendicular direction along the *y*′-axis. Hence, the *x*′- and *y*′-components of THG emission power are filtered out by introducing an analyzer orientated in parallel and perpendicular to the crystal’s optical axis.

Figure 5d–g show the measured polarization-resolved THG emission power as per the incident pump beam polarization angle for four different flakes with thicknesses of 9, 24, 36, and 61 nm. The THG emission response is highly anisotropic with four-lobe patterns. The primary maxima of THG emission are collected along around − 40° and 140° which are almost along the optical axis in the [$\overline{1 }$10] lattice direction of teallite crystal, which is consistent with the case of the measured linear optical absorption pattern, while the secondary maxima are recorded along its perpendicular direction at around 50° and 230°. The experimentally measured data points for *x*′-component, *y*′-component, and total THG emission power are shown with red squares, blue circles and black triangles, whereas the theoretical fits are shown with solid lines in the respective colors. We further corroborate the observed anisotropic THG emission with a theoretical nonlinear susceptibility model. Ternary teallite belongs to orthorhombic crystal family, therefore the contracted form of the third-order nonlinear susceptibility tensor can be written as^45, 46^

4 $${\chi }^{(3) }=\left[\begin{array}{ccc}{\chi }_{11}& 0& 0\\ 0& {\chi }_{22}& 0\\ 0& 0& {\chi }_{33}\end{array} \begin{array}{ccc}0& 0& {\chi }_{16}\\ {\chi }_{24}& 0& 0\\ 0& {\chi }_{35}& 0\end{array} \begin{array}{ccc}0& {\chi }_{18}& 0\\ 0& 0& {\chi }_{29}\\ {\chi }_{37}& 0& 0\end{array} \begin{array}{c}0\\ 0\\ 0\end{array} \right]$$

where the first term in subscript 1, 2 and 3 denotes *x*′, *y*′ and *z*′ respectively and the second subscript refers to the combination of three components as

$$\begin{array}{ccc}x^{\prime}x^{\prime}x^{\prime}& y^{\prime}y^{\prime}y^{\prime}& z^{\prime}z^{\prime}z^{\prime}\\ 1& 2& 3\end{array} \begin{array}{ccc}y^{\prime}z^{\prime}z^{\prime}& y^{\prime}y^{\prime}z^{\prime}& x^{\prime}z^{\prime}z^{\prime}\\ 4& 5& 6\end{array} \begin{array}{ccc}x^{\prime}x^{\prime}z^{\prime}& x^{\prime}y^{\prime}y^{\prime}& x^{\prime}x^{\prime}y^{\prime}\\ 7& 8& 9\end{array} \begin{array}{c}x^{\prime}y^{\prime}z^{\prime}\\ 0\end{array}$$

As per the experimental condition, teallite crystals are probed with linearly polarized electric field of $\overrightarrow{E}=\widehat{x^{\prime}}(|E|\cos\theta )+\widehat{y^{\prime}}(|E|\sin\theta )$, where $\theta$ is the linear polarization angle relative to the crystal’s optical axis. As the polarization resides in the *x′-y′* plane only, no significant contribution from the *z′*-components is accounted. Hence, the nonlinear susceptibility tensor elements containing *z′*-components can be neglected, and the resultant the electric field components of THG emission and THG intensity components can then be expressed as^12^

5 $${E}^{(3\omega )}= \left[\begin{array}{c}{E}_{x^\prime}^{(3\omega )}\\ {E}_{y^\prime}^{(3\omega )}\\ {E}_{z^\prime}^{(3\omega )}\end{array}\right] \propto {\varepsilon }_{0}{E}^{3}\left[\begin{array}{c}{\chi }_{11} {\cos}^{3}\theta + 3{\chi }_{18} \cos\theta {\sin}^{2}\theta \\ {\chi }_{22} {\sin}^{3}\theta + 3{\chi }_{29} \sin\theta {\cos}^{2}\theta \\ 0\end{array}\right]$$

6 $${I}_{x^{\prime}}^{(3\omega ) }\propto {\left({\chi }_{11} {\cos}^{3}\theta + 3{\chi }_{18} \cos\theta {\sin}^{2}\theta \right)}^{2}$$

7 $${I}_{y^{\prime}}^{(3\omega ) }\propto {\left({\chi }_{22} {\sin}^{3}\theta + 3{\chi }_{29} \sin\theta {\cos}^{2}\theta \right)}^{2}$$

The theoretical fits from Eqs. (6) and (7) are plotted in the respective colors, showing a good agreement with the measured data. Further, the theoretical fits also enable us to retrieve the relative magnitudes of the nonlinear susceptibility tensor elements ${\chi }_{11}$, ${\chi }_{18}$, ${\chi }_{22}$ and ${\chi }_{29}$. The average relative magnitudes for teallite flakes of different thicknesses are found as ${\chi }_{11}: {\chi }_{18}:{{\chi }_{22}:\chi }_{29}=1:0.037:0.86:0.029$. Also, the average value of THG anisotropic ratio ${{|\chi }_{11}|}^{2}/{{|\chi }_{22}|}^{2}$ is extracted as 1.35. Finally, the third-order nonlinear susceptibility ${\chi }^{(3)}$ value of teallite crystal is estimated by measuring the thickness-dependent THG emission. Figure 5h shows the recorded THG emission power as a function of the flake thickness. The average pump power is kept at 1.1 mW with a peak irradiance of 8.65 GW/cm^2^. Interestingly, the THG emission power gradually increases up to 4.3 pW for the 61 nm-thick flake and afterwards strong optical absorption starts playing a crucial role in attenuating the THG signal propagation so that the THG emission exponentially decays for the relatively thicker teallite flakes. In fact, the exponential decay of THG emission enables us to extract the imaginary part of refractive index (*k*_*3*_) at emission wavelength (${\lambda }_{3}$ = 520 nm) by fitting the thickness-dependent THG emission power equation ${P}^{\left(3\omega \right)}\left(l\right)=A{d}^{2}{\exp}\left(-\frac{4\pi {k}_{3}d}{{\lambda }_{3}}\right)$, where $d$ is thickness of flake and $A$ is a constant. Figure 5h shows the corresponding plot with the measured data (black squares) and the fitted curve (green curve) for *k*_*3*_ = 1.42. We assume the refractive index of teallite is similar to that of herzenbergite, where *n*_*3*_ = 4.39 at 520 nm and *n*_*1*_ = 3.61 at 1560 nm along the armchair direction^47^. Thus, taking these values along with other experimental parameters into account (${P}^{\left(\omega \right)}$ = 1.1 mW, laser pulse width *τ* = 90 fs, repetition rate ${f}_{rep}$ = 80 MHz, and spot size *W* = 1.5 µm at the fundamental wavelength ${\lambda }_{1}$ = 1560 nm), the magnitude of ${\chi }^{(3)}$ can be estimated by the following formula^11^

8 $$\left|{\chi }^{\left(3\right)}\right|= {\left[\frac{16\sqrt{{n}_{3}^{2}+{k}_{3}^{2}}{n}_{1}^{3}{\epsilon }_{0}^{2}{c}^{4}{f}_{rep}^{2}{W}^{4}{\tau }^{2}{\left[\frac{\pi }{4{\ln}2}\right]}^{3}{P}^{\left(3\omega \right)}}{9{\omega }^{2}{d}^{2}{{P}^{\left(\omega \right)}}^{3}}\left(\frac{\left(\frac{4{\pi }^{2}{k}_{3}^{2}{d}^{2}}{{\lambda }_{3}^{2}} + {\Delta k}^{2}{d}^{2}\right)}{{e}^{-\frac{4\pi {k}_{3}d}{{\lambda }_{3}}}-2{{\cos}(\Delta kd)e}^{-\frac{2\pi {k}_{3}d}{{\lambda }_{3}}}+1}\right){e}^{\frac{4\pi {k}_{3}d}{{\lambda }_{3}}}\right]}^{1/2}$$

where *n*_*1*_ and *n*_*3*_ are the real part of refractive index at pump wavelength (${\lambda }_{1}$) and emission wavelength (${\lambda }_{3}$), $\Delta k= \frac{6\pi }{{\lambda }_{1 }} ({n}_{1}- {n}_{3})$ is the phase mismatch between the fundamental beam and the forward propagating THG emission beam in the transmission optical setup arrangement. Hence, the magnitude of ${\chi }^{(3)}$ for teallite crystal is estimated as 3.49 × 10^–19^ m^2^/V^2^, which is within same order of magnitude as the previously reported 2D layered materials.

## Discussion

To conclude, we have demonstrated the polarization-dependent anisotropic optical responses from the mechanically exfoliated teallite flakes of various thicknesses. The exfoliated ternary teallite crystals are investigated using HRTEM and EDXS characterization tools to determine the structural and chemical composition. Further, the anisotropic Raman vibrational modes and linear optical properties are probed with polarization-resolved Raman spectroscopy and optical absorption spectroscopy, which determine the armchair direction and the optical axis of teallite crystals, respectively. In addition, teallite crystals are found to exhibit strong modulation of direct optical band gap depending on the incident linear polarization angle. Finally, we investigate anisotropic nonlinear optical properties of teallite crystals with the THG emission measurement and estimate the third-order nonlinear susceptibility value. Our findings also emphasize on the distorted lattice symmetry induced linear and nonlinear optical responses in teallite crystals, due to the element alloying in complex multi-element layered materials. In hindsight, we envisage that these outcomes would lead to a better understanding of the anisotropic optical responses in natural multi-element layered materials and can have implications in optical strain sensors, photodetectors, frequency modulators, encrypted signal processing, and other prototype nonlinear photonic applications.

## Methods

### Sample preparation

Teallite flakes of various thicknesses are mechanically exfoliated from bulk natural teallite mineral (from Monserrat, Oruro, Bolivia) using Nitto tape (SPV 224) and Scotch tape. The glass substrates with 1 cm × 1 cm are treated with acetone, deionized water and isopropanol followed by ultra-sonication for 20 min to remove the undesirable residues from the surface. These pretreated glass substrates are used to transfer the mechanically exfoliated flakes.

### Polarization-resolved Raman spectroscopy

The sample is illuminated with a 632.8 nm He–Ne laser using a 40× objective lens (NA = 0.65) and the back-reflected signal is collected via the same objective lens to a spectrometer (Horiba, iHR 520) with a beam splitter and sets of mirrors. The elastic scattered light is rejected using an edge filter (Semrock, LP02-633RE-25) in the collection path. The desired linear polarization of excitation beam is obtained using a combination of a linear polarizer and a rotating half-wave plate in the illumination path. The collected signal is further passed through a linear polarization analyzer for resolving the parallel polarization component of the Raman spectra.

### Polarization-resolved optical absorption spectroscopy

To record the polarization‐resolved optical absorption spectra, a broadband white light source (Thorlabs, SLS201L, 360–2600 nm) is passed through a combination of a linear polarizer and half-wave plate and then focused on the probed sample with a 80× objective lens (NA = 0.5). To measure the reflection spectrum, the back‐reflected light is collected from the sample using the same objective lens and routed towards the spectrometer using a beam splitter and sets of mirrors, whereas in the case of the transmission spectrum, the transmitted light through the sample is collected using another 100× objective lens (NA = 0.7) and routed to the spectrometer using mirrors. Further, the reflection and transmission spectra are normalized with the light source spectrum to achieve the reflectance (*R*) and transmittance (*T*) spectra. Finally, the absorbance (*A*) spectrum is obtained by using the relation of *A* = 1 – *R* – *T*. We also estimate the measurement uncertainties in transmittance, reflectance, absorbance, and optical band gap. To perform this, the transmittance (*T*) and reflectance (*R*) spectra are recorded multiple times. The average values of these spectra in the respective configuration ${T}_{avg}$ and ${R}_{avg}$ are obtained, and the measurement errors of transmittance and reflectance $\delta T$ and $\delta R$ are approximated as the difference of maximum and minimum counts at each wavelength. Then the measurement uncertainties in transmittance and reflectance are estimated as $\delta T/{T}_{avg}$ and $\delta R/{R}_{avg}$, and the uncertainty in absorbance is obtained as $\delta A/A$ = $\delta T/{T}_{avg}$  + $\delta R/{R}_{avg}$. Further, the uncertainties in transmittance and reflectance are used to estimate the uncertainty in absorption coefficient $\delta \alpha /\alpha$ by using Eq. (3), and subsequently the uncertainty in optical band gap is estimated as $\delta {E}_{g}/{E}_{g}$ = $2\cdot \delta \alpha /\alpha$. According to our analysis, the measurement uncertainties in transmittance, reflectance and absorbance turn out to be around 0.14% (0.20%), 0.22% (0.31%), and 0.36% (0.51%) at the wavelength of 520 nm (720 nm). The estimated uncertainty in optical band gap measurement is then around 0.66%.

### THG emission measurement

The sample is pumped with a femtosecond laser source at the wavelength of 1560 nm (pulse width 90 fs, repetition rate 80 MHz) using a 40× objective lens (NA = 0.65). The transmitted THG signal is collected through a 100× objective lens (NA = 0.7) and routed towards the imaging camera and the spectrometer (Horiba, iHR 520) for recording the THG image and the corresponding spectra, respectively. To reject the incident pump beam, the collected signal is passed through a shortpass filter in the collection path.
